# A CD25–chemokine receptor complex initiates noncanonical IL-2 signaling

**DOI:** 10.1016/j.jbc.2025.110981

**Published:** 2025-11-25

**Authors:** Ho-Sup Lee, Sarah Hyun Ji Kim, Javid Aceil, Amelia Meecham, Alexandre Gingras, Klaus Ley, Jamie B. Spangler, Mark H. Ginsberg

**Affiliations:** 1 Department of Medicine, University of California San Diego School of Medicine, La Jolla, California, USA; 2 Department of Physiology, Augusta University, Augusta, Georgia, USA; 3 Department of Biomedical Engineering, Johns Hopkins University, Baltimore, Maryland, USA; 4 Department of Chemical & Biomolecular Engineering, Johns Hopkins University, Baltimore, Maryland, USA

**Keywords:** interleukin 2, integrin, heparan sulfate, T-cell biology, signaling, chemokine receptor, CCR7, CCR5, CXCR4, CD25

## Abstract

An antimouse CD25 antibody, PC61, induces a complex formed by the interleukin-2 (IL-2)-dependent association of CD25 with CCR7 and an alternative IL-2 signaling pathway that results in integrin activation in CD4^+^CD25^Hi^Foxp3^+^ regulatory T cells (Tregs). Here, we used structure-based design together with combinatorial screening to identify a human IL-2 mutant (IL-2(E52K)) that disrupts CD25–CCR7 complex formation while retaining the full CD25 affinity of the parent molecule. An anti–human CD25 (hCD25), 7G7B6, drove formation of IL-2-dependent hCD25–CCR7, CD25–CXCR4, and CD25–CCR5 complexes and induced integrin activation in hCD25-expressing IL-2Rα^+^ YT-1 cells, Jurkat T cells, and primary Tregs. IL-2(E52K) failed to support activation in CCR5^Lo^ Jurkat T cells and primary Tregs. In contrast, IL-2(E52K) supported activation in CCR5^Hi^ IL-2Rα^+^ YT-1 cells, which was blocked by the CCR5-specific antagonist, maraviroc. Heparan sulfate (HS), a physiological ligand of IL-2, induced IL-2-dependent CD25–CCR7 association, and IL-2(E52K) failed to support HS-induced CD25–CCR7 complex formation and integrin activation in Jurkat cells. Both HS and 7G7B6 did not block canonical IL-2 signaling. CD122 was present in the 7G7B6-induced CCR7–CD25 complex. CD122 forms a heterodimer with CD132 (the common γ chain) that triggers canonical IL-2 signaling. Thus, both anti-CD25 antibody and HS require formation of a chemokine receptor–CD25 complex to initiate alternative IL-2 signaling. In addition, our findings suggest that alternative and canonical IL-2 signaling receptors can be incorporated into the same multiprotein assembly, allowing for a single complex to mediate divergent effects on downstream signaling.

Interleukin-2 (IL-2) controls the expansion and maintenance of T-cell subsets. IL-2 binds to the α receptor (CD25), encoded by *IL2RA*, to promote IL-2 association with the signaling β (CD122) and γ (CD132) subunits; the resulting trimeric receptor triggers canonical signaling *via* associated Janus kinases (JAKs) 1 and 3 that phosphorylate signal transducer and activator of transcription 5 (STAT5) (1, 2). IL-2 also signals through a heterodimeric complex comprising the β and γ subunits, albeit with a 100-fold lower affinity (3). CD4^+^FOXP3^+^CD25^Hi^ regulatory T cells (Tregs), which provide a critical brake on immune responses and inflammation (4), are dependent on IL-2 and integrins. Dynamic increases in integrin affinity for macromolecular ligands (5) (“activation”) are essential for the function and phenotype of Tregs (6). Thus, augmenting integrin activation in Tregs may enhance suppressive function and blunt autoimmunity and inflammation.

PC61 (7), in contrast with some other antimouse CD25 antibodies, induces IL-2-dependent integrin activation in murine Tregs and amplifies their suppressive function (8). Both these functional effects of PC61 require Rap1, a signal transducer that drives integrin activation in Tregs (8, 9). Because CD25 lacks endogenous signaling capability, PC61 binding must engage a “signaling” receptor; however, PC61 does not trigger or augment STAT5 phosphorylation (8). Furthermore, PC61-induced integrin activation proceeds in the absence of CD122 or in the presence of blocking anti-CD122 antibody or a JAK 1,3 inhibitor (8); thereby indicating that canonical IL-2 signaling is not involved. Pertussis toxin, an inhibitor of Gαi (10), blocks PC61-induced integrin activation. Heparan sulfate (HS) docks IL-2 in tissues (11) and can promote Treg-suppressive function (12, 13). HS also promotes pertussis toxin–inhibitable IL-2-induced integrin activation (8), suggesting that it might trigger this same alternative IL-2 signaling pathway. We termed this alternative IL-2 signaling pathway monoclonal antibody (or matrix)–directed alternative cytokine signaling (MACS) (8). PC61 induced the formation of a complex containing CD25, IL-2, and CCR7. Because chemokine receptors signal *via* Gαi (14), we hypothesized that formation of this complex initiates MACS.

Here, we establish the mechanism for triggering MACS by using structure-based design coupled with combinatorial screening to identify an IL-2 mutant (IL-2(E52K)) that does not support formation of the CD25–IL-2–CCR7 complex. We show that 7G7B6, an anti–human CD25 (hCD25), induces assembly of an IL-2-dependent hCD25–CCR7 complex. IL-2(E52K) did not support 7G7B6-induced CD25–CCR7 complex formation. 7G7B6 triggered IL-2-dependent integrin activation in CD25-expressing Jurkat human T cells and primary human Tregs. IL-2(E52K) lacked the capacity to support 7G7B6-induced integrin activation in these cells, thereby tying formation of a CD25–IL-2–CCR7 complex to MACS. IL-2 and IL-2(E52K) induced STAT5 phosphorylation with similar potency, indicating that the mutation did not disrupt canonical IL-2 signaling. Thus, formation of a CD25–IL-2–CCR7 complex initiates MACS, and an IL-2 mutation can dissociate these two signaling pathways.

We report that HS induced IL-2-dependent CD25–CCR7 association and integrin activation that was lost with IL-2(E52K), thereby establishing that formation of an HS-induced IL-2-dependent chemokine receptor CD25 complex also triggers MACS. Neither 7G7B6 nor HS inhibited IL-2-induced STAT5 phosphorylation, indicating that MACS and canonical IL-2 signaling pathways can operate concomitantly. Indeed, we report that 7G7B6 induces the formation of an IL-2-dependent multiprotein complex that incorporates both CCR7 and CD122, receptors that trigger MACS or canonical IL-2 signaling, respectively. In sum, these data establish that formation of CD25–IL-2–chemokine receptor complex triggers MACS in humans, show that certain anti-CD25 antibodies and HS induce MACS through common features of IL-2, and propose that both canonical and MACS IL-2 signals can emanate from the same multiprotein complex.

## Results

### IL-2 promotes interaction of murine CD25 with human CCR7

**Figure 1:**
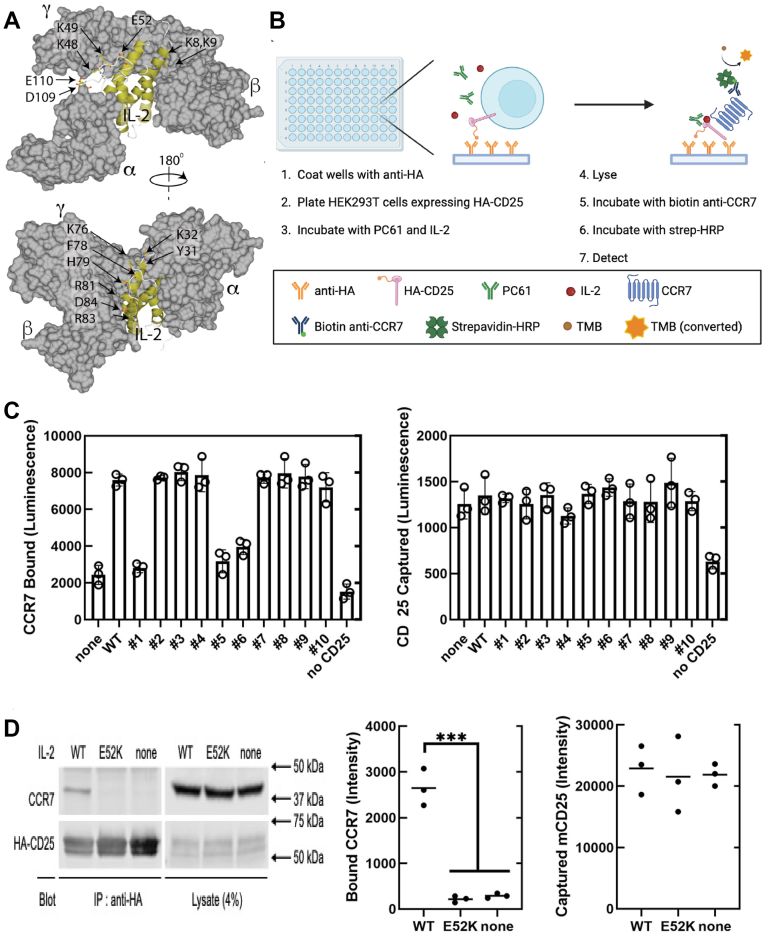
**ELISA screen identifies an IL-2 mutant, IL-2(E52K), that does not support the CD25–CCR7 complex.***A,* design strategy. Depicted are space-filling models of the human trimeric IL-2 receptor (*gray*) with the helical elements of IL-2 depicted as a *gold ribbon*. Charged residues in unburied IL-2 loops are labeled and targeted for mutagenesis. *B,* schematic of ELISA. HEK293T-HA-murine CD25 (mCD25) cells were plated on anti-HA–coated wells and then incubated with 1 μg/ml IL-2 (*red*) and 5 μg/ml PC61 (*green*) for 1 h at 37 °C. Cells were lysed, and washed plates were probed by anti-CCR7 or anti-HA, followed by chemiluminescence reagents. *C,* ELISA screen identifies mutants that fail to support the CD25–CCR7 complex. Note that CCR7 association is IL-2 dependent and reduced in mutants 1, 5, and 6 (mean ± SEM; N = 3). *D,* IL-2(E52K) fails to support the CD25-CCR7 complex. The experiment was performed as in *B* and *C* with detection by immunoprecipitation of HA-mCD25 followed by immunoblotting. Scatter plots on the *right* show the mean (horizontal line) quantified intensities of CCR7 and HA-mCD25 from three independent experiments ns, not significant, ∗∗∗0.001 < *p* < 0.01 by unpaired *t* tests. HA, hemagglutinin; HEK293T, human embryonic kidney 293T cell line; IL, interleukin.

To test the hypothesis that the formation of a CD25–CCR7 complex triggers MACS, we sought IL-2 mutants that prevent the formation of this complex but do not disrupt other critical receptor interactions. IL-2 is required for the formation of the complex, and mutation of the IL-2 binding site of CD25 or anti-CD25 antibodies that block IL-2 binding prevents complex formation (8). Thus, we reasoned, by analogy to IL-2-mediated assembly of the trimeric receptor (15), that PC61 might cause CD25-bound IL-2 to interact with CCR7 to initiate MACS. We used the structure of trimeric human receptor–bound IL-2 (15) to create IL-2 mutants that might not disrupt interaction with the αβγ receptor. We selected charge reversal substitutions in unstructured loops to minimize potential protein misfolding (Fig. 1*A*). With these considerations, we constructed human IL-2 mutations (Table 1).

**Table 1:** Mutations in human IL-2

Mutant #	Mutated residue	Mutation site with flanking sequence
1	Q22V, Q126A, I129D, S130G	LDL**V**MIL, TFC**A**SI**DG**TLT
2	Y31A, K32E	INN**AE**NPK
3	K76E, F78A, H79A	AQS**E**N**AA**LRP
4	K8D, K9D	SST**DD**TQL
5	K48D, K49D	YMP**DD**ATE
6	E52K	KAT**K**LKH
7	D109K, E110K	EYA**KK**TAT
8	R81E	FHL**E**PRD
9	D84K	RPR**K**LIS
10	R83E	LRP**E**DLI

IL-2 mutants and the sites of mutated amino acid residues. Mutations are provided in bold text.

An ELISA measured the association of CD25 and CCR7 with detection by chemiluminescence (Fig. 1*B*). Human embryonic kidney 293T (HEK293T) cells expressing hemagglutinin (HA)-tagged murine CD25 (mCD25) were plated on anti-HA–coated plates and incubated with PC61 and IL-2, followed by cell lysis and measurement of bound CCR7 and CD25. The increase in luminescence when both IL-2 and PC61 were added to HA-mCD25–expressing cells revealed that PC61 induced IL-2-dependent CD25–CCR7 association, whereas this increased luminescence was not observed with the addition of PC61 or IL-2 alone. IL-2 mutants 1, 5, and 6 showed a twofold reduction in luminescence (Fig. 1*C*); we selected mutant 6 (IL-2(E52K)) for further study because mutant 1 is known to block IL-2Rγ subunit interaction (16), and recombinant mutant 5 was not well secreted. Coimmunoprecipitation of CCR7 and mCD25 confirmed that IL-2, but not IL-2(E52K), enabled PC61-induced CD25–CCR7 association (Fig. 1*D*).

**Figure 2:**
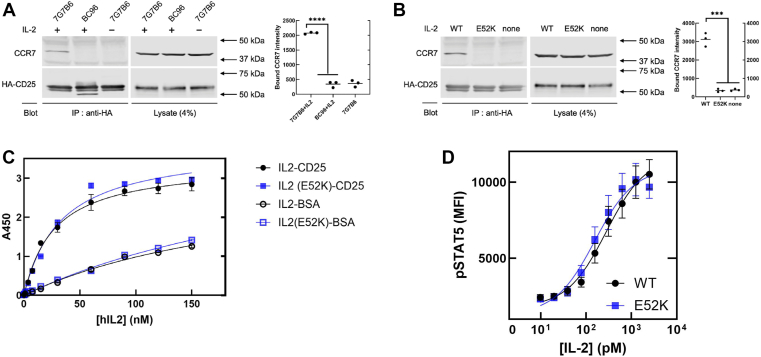
**IL-2(E52K) does not support the assembly of the hCD25–CCR7 complex but supports canonical IL-2 signaling.***A,* 7G7B6 triggers IL-2-dependent hCD25–CCR7 complex. Anti-hCD25 antibodies (5 μg/ml), 7G7B6 or BC96, were incubated with HA-hCD25–expressing HEK293T cells in the presence or the absence of 1 μg/ml IL-2 and following lysis and immunoprecipitation with anti-HA were assayed for captured CCR7 or HA-hCD25 by immunoblotting. Scatter plots on the *right* show the mean (horizontal line) quantified intensities of CCR7 and HA-hCD25 from three independent experiments. *B,* IL-2(E52K) does not support the 7G7B6-induced the hCD25–CCR7 complex. IL-2(E52K) or wildtype IL-2 was assayed for capacity to support the 7G7B6-induced CD25–CCR7 complex as described in *A*. Scatter plots on the *right* show the mean (horizontal line) quantified intensities of CCR7 and CD25 from three independent experiments. *C,* IL2(E52K) mutation does not reduce binding of IL-2 to hCD25. Microplates coated with recombinant hCD25 extracellular domain or BSA were incubated with the indicated concentrations of IL-2 WT or IL-2(E52K), and bound IL-2 was quantified by ELISA. *D,* IL-2(E52K) triggers canonical IL-2 signaling. IL2Rα^+^ YT-1 cells were stimulated with varying concentrations of IL-2 WT or IL-2(E52K) (for 30 min at 37 °C, followed by staining with APC-anti–phospho-STAT5 and analysis by flow cytometry (mean ± SEM; N = 3). ns, not significant, ∗∗∗*p* < 0.0001, ∗∗∗∗*p* < 0.0001 by one-way ANOVA. BSA, bovine serum albumin; HA, hemagglutinin; hCD25, human CD25; HEK293T, human embryonic kidney 293T cell line; IL, interleukin; STAT5, signal transducer and activator of transcription 5.

We used HEK293T cells expressing hCD25 to test for commercial anti-hCD25 antibodies that drive CD25–CCR7 association. We found that 7G7B6 (17), but not BC96, induced IL-2-dependent hCD25–CCR7 association (Fig. 2*A*). Importantly, the precise epitopes of PC61 on mCD25 or 7G7B6 on hCD25 have not been mapped. Nevertheless, as is true of PC61, IL-2(E52K) failed to support the 7G7B6-induced hCD25–CCR7 complex (Fig. 2*B*). IL-2(E52K) neither impaired IL-2 binding to hCD25 (Fig. 2*C*) nor reduced IL-2-driven STAT5 phosphorylation (Fig. 2*D*, Supporting Information Fig. S1). Thus, specific features of IL-2, distinct from those required for canonical IL-2 signaling, promote association of CD25 with CCR7 in the presence of certain anti-CD25 antibodies.

### Many chemokine receptors can associate with CD25 in the presence of IL-2 and some anti-CD25 antibodies

**Figure 3:**
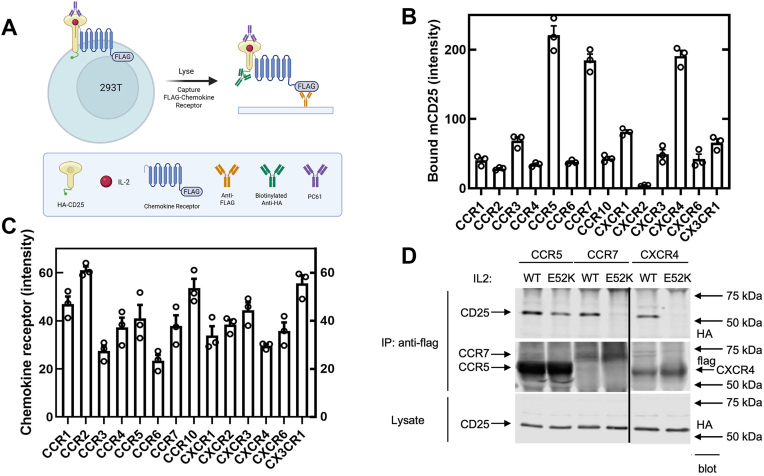
**Human CXCR4 or CCR5 can form a complex with hCD25.***A*, schematic of the assay. HEK293T-HA-mCD25 cells were transfected with cDNA encoding the indicated FLAG-tagged murine chemokine receptor and then incubated with 1 μg/ml IL-2 (*red*) and 5 μg/ml PC61 (*purple*) for 1 h at 37 °C. Cells were lysed, and immunoprecipitates were fractionated by SDS-PAGE followed by immunoblotting for HA-mCD25 (*B*) or FLAG-murine chemokine receptor (*C*). Bar graphs depict quantified intensities of HA-mCD25 or FLAG-chemokine receptors from three independent experiments (mean ± SEM; N = 3). *D,* IL-2(E52K) supports the hCD25–CCR5 complex assembly. The experiment was performed as in *B* and *C* with HEK293T cells expressing HA-hCD25, human chemokine receptors, and the use of 7G7B6 in place of PC61. FLAG-human chemokine receptor was immunoprecipitated, followed by fractionation by SDS-PAGE and immunoblotting for HA-hCD25 or FLAG-chemokine receptor. The *black vertical line* indicates the site at which two irrelevant lanes were removed, and the remaining lanes were joined. cDNA, complementary DNA; HA, hemagglutinin; hCD25, human CD25; HEK293T, human embryonic kidney 293T cell line; IL-2, interleukin 2; mCD25, murine CD25.

We previously observed that silencing CXCR4 could inhibit MACS signaling (8), suggesting that chemokine receptors other than CCR7 could mediate MACS. To test which chemokine receptors might support MACS, we transiently transfected HA-tagged mCD25-expressing HEK293T cells with each of several FLAG-tagged murine chemokine receptors and incubated them with PC61 in the presence of IL-2 (Fig. 3*A*). After lysis, anti-FLAG immunoprecipitates were fractionated by SDS-PAGE followed by immunoblotting for HA-mCD25 (Fig. 3*B*) or FLAG-chemokine receptor (Fig. 3*C*). HA-mCD25 was abundant in CCR7, CXCR4, and CCR5 immunoprecipitates (Fig. 3*B*) relative to other receptors that were also well expressed (*e.g.*, CCR2, CCR10, CX3CR1) (Fig. 3*C*). We then confirmed that human CCR7, CXCR4, and CCR5 would form a complex with hCD25 in the presence of 7G7B6 (Fig. 3*D*). Notably, unlike CCR7 and CXCR4, CCR5 formed a complex with hCD25 in the presence of IL-2(E52K) and 7G7B6 (Fig. 3*D*).

### IL-2(E52K) blocks MACS-dependent integrin activation

**Figure 4:**
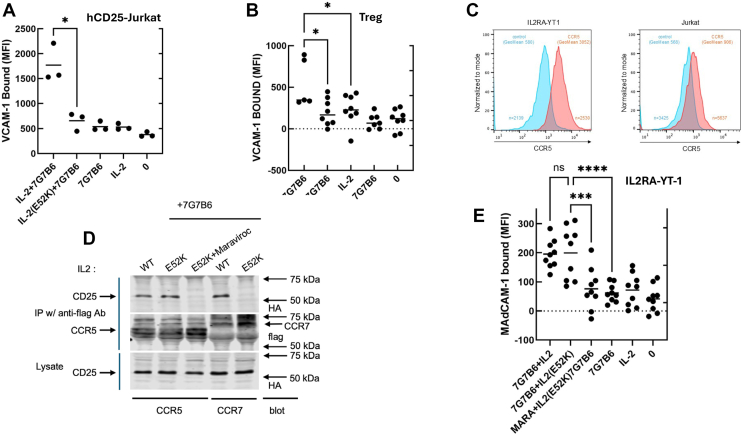
**hCD25–chemokine receptor complex triggers integrin activation.***A* and *B,* IL-2(E52K) does not trigger MACS-dependent integrin activation in a CD25-expressing T-cell line or Tregs. *A,* hCD25-Jurkat T cells or (*B*) primary human Tregs were incubated with 5 μg/ml VCAM-1 Fc (these cells express primarily α4β1 rather than α4β7) and 5 μg/ml 7G7B6 or isotype control in the presence or the absence of 1 μg/ml IL-2(WT) or IL-2(E52K) for 30 min at 37 °C before measurement of bound VCAM-1 Fc by flow cytometry. C, IL2RA^+^-YT1 cells express ∼8-fold more CCR5 than hCD25 Jurkat T cells. Depicted are flow cytometry histograms and geometric mean fluorescence intensity of staining with anti-CCR5 or isotype control antibody. *D,* a CCR5 antagonist blocks assembly of the 7G7B6-induced CD25–CCR5 complex. HEK293T-HA-hCD25 cells were transfected with cDNA encoding the indicated FLAG-tagged chemokine receptor and then incubated with 5 μg/ml 7G7B6 and 1 μg/ml IL-2 (WT), IL-2(E52K), or IL-2(E52K)+ 50 nM maraviroc for 1 h at 37 °C. Cells were lysed, and immunoprecipitates were fractionated by SDS-PAGE followed by immunoblotting for HA-hCD25 (*E*) 7G7B6-induced the CCR5–CD25 complex triggers integrin activation. IL2RA^+^-YT1 cells (express α4β7) were incubated with 5 μg/ml MAdCAM-1 Fc in the presence or the absence of 5 μg/ml 7G7B6 and/or isotype control (0) in the presence or the absence of 1 μg/ml IL-2, IL-2(E52K), or 50 nM maraviroc (Mara) + IL-2(E52K) for 30 min at 37 °C before measurement of bound VCAM-1 Fc by flow cytometry: ∗*p* < 0.05, ∗∗ *p* < 0.01, by one-way ANOVA with Tukey’s correction, Scatter plots depict mean (horizontal line) from at least three independent experiments. cDNA, complementary DNA; HA, hemagglutinin; hCD25, human CD25; HEK293T, human embryonic kidney 293T cell line; IL-2, interleukin 2; Treg, regulatory T cell; VCAM-1, vascular cell adhesion molecule 1.

In the presence of wildtype IL-2, 7G7B6 stimulated increased vascular cell adhesion molecule 1 (VCAM-1) binding, a measure of activation of integrin α4β1, to hCD25-expressing Jurkat T cells (Fig. 4A). In sharp contrast, IL-2(E52K) failed to support 7G7B6-induced α4β1 activation. These results were recapitulated with primary hCD25^Hi^CD127^Lo^ Tregs, which are predominantly CCR5^Lo^ (18), wherein wildtype IL-2, but not IL-2(E52K), supported 7G7B6-induced integrin activation (Fig. 4*B*). In initial experiments with an immortalized CD25^Hi^ NK cell line, IL-2Rα^+^ YT-1 (19, 20), 7G7B6 increased MAdCAM-1 binding (these cells express integrin α4β7) in the presence of IL-2 and not IL-2(E52K); however, after several months in continuous culture, they became responsive to IL-2(E52K). We hypothesized that this change was due to the emergence of a chemokine receptor that could engage IL-2(E52K)-bound CD25. We had found (Fig. 3*D*) that CCR5 was such a receptor, and our IL-2Rα^+^ YT-1 cells presently express much more CCR5 than the Jurkat cells (Fig. 4*C*). Addition of 50 nM maraviroc, a CCR5 antagonist (21), blocked the CD25–CCR5 complex formed in HEK293T cells in the presence of 7G7B6 and IL-2(E52K) (Fig. 4*D*). Furthermore, maraviroc blocked 7G7B6-induced MAdCAM1 binding to IL-2Rα^+^ YT-1 cells in the presence of IL-2(E52K) (Fig. 4*E*), confirming that IL-2-mediated association of CD25 with CCR5 can initiate MACS and explaining the emergence of responsiveness to IL-2(E52K) in these IL-2Rα^+^ YT-1 cells. Thus, formation of an IL-2-dependent CD25–chemokine receptor complex is required for anti-CD25–induced integrin activation.

### HS triggers IL-2-dependent interaction of CD25 and CCR7, resulting in MACS

**Figure 5:**
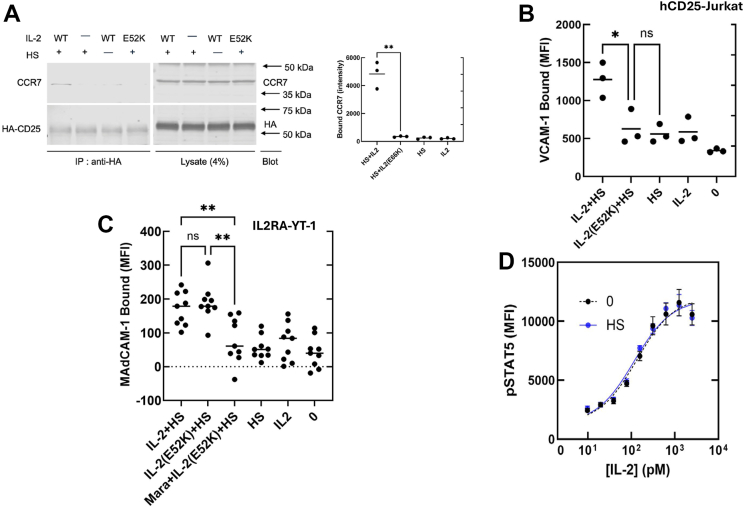
**HS induces an IL-2-dependent CD25–CCR7 complex to trigger MACS.***A,* IL-2(E52K) does not support HS-induced CD25–CCR7 association. Membrane lysates from HA-hCD25–expressing HEK293T cells were incubated with 5 μg/ml HS in the presence (+) or absence (−) of 2.5 μg/ml IL-2 (WT) or IL-2 (E52K) for 30 min at 37 °C. Anti-HA immunoprecipitates were fractionated by SDS-PAGE, and blots were probed with either anti-HA or anti-CCR7. Quantification of data by LI-COR infrared spectroscopy from three independent experiments is shown to the *righ*t of the blot. *B,* IL-2(E52K) does not support HS-induced integrin activation. hCD25-Jurkat cells were incubated with 5 μg/ml HS in the presence or the absence (−) of 2.5 μg/ml IL2 (WT) or IL-2(E52K) and 5 μg/ml VCAM-1 for 30 min at 37 °C, and bound VCAM-1 was measured by flow cytometry. *C,* HS-induced CCR5–CD25 complex triggers integrin activation. IL2RA^+^-YT1 cells were incubated with 5 μg/ml MAdCAM-1 Fc in the presence or the absence of 40 μg/ml HS and/or buffer control (0) in the presence or the absence of 1 μg/ml IL-2, IL-2(E52K), 50 nM maraviroc (Mara) + IL-2(E52K) for 30 min at 37 °C before measurement of bound MAdCAM-1 Fc by flow cytometry. *D,* HS did not affect canonical IL-2 signaling. IL-2Rα^+^ YT-1 cells were incubated with varying concentrations of IL-2 WT in the presence (*blue*) or the absence (0, *black*) of 40 μg/ml HS for 30 min at 37 ^o^C followed by analysis of phospho-STAT5 staining by flow cytometry. Curves were fitted to a simple agonist response model by nonlinear regression on GraphPad Prism, version 10. ∗*p* < 0.05, ∗∗*p* < 0.01 by unpaired *t* tests (*A*) or one-way ANOVA with Tukey’s correction (*B*, *C*), mean ± SEM from at least three independent experiments. HA, hemagglutinin; hCD25, human C25; HEK293T, human embryonic kidney 293T cell line; HS, heparan sulfate; IL-2, interleukin 2; MACS, monoclonal antibody (or matrix)–directed alternative cytokine signalling; STAT5, signal transducer and activator of transcription 5; VCAM-1, vascular cell adhesion molecule 1.

We previously showed that HS, a physiological ligand of IL-2, can bias IL-2 signaling toward integrin activation (8). Incubation of solubilized membranes of hCD25-expressing HEK293T cells with HS and IL-2, but not IL-2(E52K), resulted in coimmunoprecipitation of hCD25 and CCR7 (Fig. 5*A*). Furthermore, in the presence of IL-2, the addition of HS to CD25-expressing Jurkat T cells caused increased VCAM-1 binding, whereas IL-2(E52K) failed to support HS-induced VCAM-1 binding (Fig. 5*B*). In contrast, in the presence of IL-2 or IL-2(E52K), the addition of HS to IL-2Rα^+^ YT-1 cells caused increased MAdCAM-1 binding (Fig. 5*C*). Addition of maraviroc abrogated IL-2(E52K)’s capacity to support HS-induced MAdCAM-1 binding to these cells. Previous work showed that isolated CD25 or CD122 neither competed for heparin binding to IL-2 nor did heparin blocked IL-2’s capacity to support proliferation of CTLL2 cells (22). Consistent with these results, we found that 40 μg/ml HS did not block the pSTAT5 response to IL-2 (Fig. 5*D*), suggesting that HS does not interfere with IL-2 interaction with the high-affinity αβγ receptor. Thus, HS can trigger IL-2-dependent CD25–chemokine receptor association that triggers MACS without impairing canonical IL-2 signaling.

### A 7G7B6-induced IL-2-dependent complex incorporates both CCR7 and CD122

**Figure 6:**
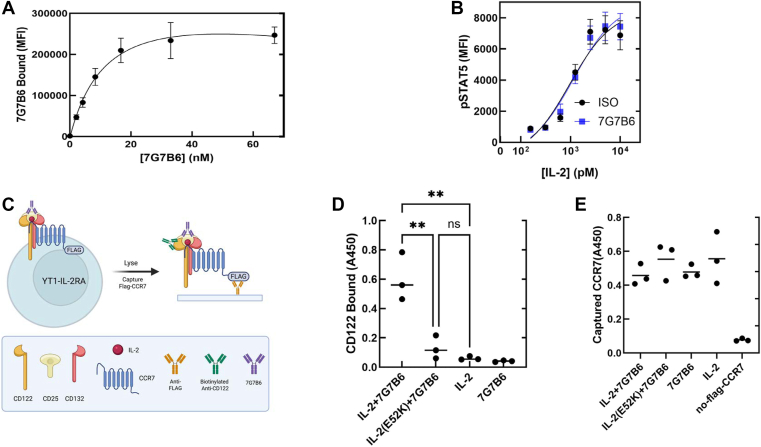
**A canonical signaling IL-2 receptor is incorporated into the CCR7–CD25 complex.***A,* Binding of 7G7B6 to IL-2RΑ^+^ YT-1 cells. The indicated concentration of 7G7B6 was incubated with IL2RA^+^-YT1 cells for 30 min at 37 °C, and bound 7G7B6 was detected with phycoerythrin–anti-mouse IgG by flow cytometry. Nonlinear least squares curve fitting to a single class of binding sites by PRISM, version 10.1. *B,* 7G7B6 does not inhibit canonical IL-2 signaling. IL2Rα^+^-YT-1 cells were stimulated with varying concentrations of IL-2 WT and 5 μg/ml of isotype (iso, *black*) or 7G7B6 (7G7B6, *red*) for 30 min at 37 °C, followed by staining with PE-anti-phospho-STAT5 and analysis by flow cytometry. *C,* Schematic of assay for incorporation of CD122 into the CD25–CCR7 complex. IL-2RΑ^+^ YT-1 cells stably expressing FLAG-CCR7 were incubated for 30 min at 37 °C with 7G7B6 (5 μg/ml) and IL-2 (1 μg/ml) or IL-2(E52K). Cells were lysed on ice, and the resulting lysates were incubated in a 96-well microplate coated with rabbit anti-FLAG antibody for 2 h at room temperature. After washing, associated CD122 or captured CCR7 were detected with biotinylated anti-CD122 or biotinylated anti-CCR7, respectively. The biotinylated antibodies were reacted with streptavidin peroxidase, followed by TMB chromogenic substrate and quantified by absorbance at 450 nm on a microplate reader. *D,* CD122 bound. “IL-2” indicates absorbance at 450 nm in the absence of 7G7B6, no-FLAG-CCR7 indicates absorbance at 450 nm when IL-2RΑ^+^ YT-1 cells lacking CCR7 were incubated in the presence of IL-2 and 7G7B6. *E,* CCR7 bound. Captured complexes were reacted with biotinylated anti-CCR7 in place of biotinylated anti-CD122. ∗∗∗0.001 < *p*, by one-way ANOVA with Tukey’s correction, mean (horizontal line) from three independent experiments. IL-2, interleukin 2; STAT5, signal transducer and activator of transcription 5; TMB, 3,3′,5,5′-tetramethylbenzidine.

7G7B6 does not block IL-2 binding to CD25-expressing cells (17) suggesting that it, like HS, might trigger MACS without impairing canonical signaling. Unlike HS, 7G7B6 is not heterogeneous, enabling us to define a saturating concentration for 7G7B6 binding to IL-2Rα^+^ YT-1 cells (Fig. 6*A*). A saturating concentration of 7G7B6 did not alter the pSTAT5 response (Fig. 6*B*). Thus, canonical and MACS IL-2 signaling can proceed concurrently, suggesting that their signal-initiating receptors, CD122 and chemokine receptor, both might incorporate into the same IL-2-dependent protein assembly. To test this idea (Fig. 6*C*), we transduced IL-2Rα^+^ YT-1 cells with FLAG-tagged CCR7 and incubated them with 33 nM 7G7B6 in the presence of IL-2 or IL-2(E52K). We lysed the cells, captured the FLAG-CCR7 in anti-FLAG–coated ELISA wells, and detected captured CD122 with biotinylated anti-CD122. CD122 was associated with the captured CCR7 in the presence of IL-2 but not IL-2(E52K). Furthermore, the association of CD122 with CCR7 was not induced by IL-2 in the absence of 7G7B6 (Fig. 6*D*). Similar quantities of CCR7 were captured in the presence or the absence of IL-2 or 7G7B6 (E). Thus, in the presence of 7G7B7, IL-2 can support the association of CD122 with CCR7, suggesting that CCR7 and CD122 can signal in tandem to initiate coincident MACS and canonical IL-2 signaling.

## Discussion

IL-2 signaling *via* the αβγ receptor shapes immune responses by promoting the proliferation of T cells and, in particular, enabling the development, function, and stability of Tregs (1). IL-2 can also signal *via* an alternative MACS pathway to promote Treg-suppressive function (8). Here, we used the structure of IL-2 bound to the canonical αβγ receptor to construct an IL-2 mutant (IL-2(E52K)) that fails to support an anti-hCD25 (7G7B6)-induced CD25–CCR7 complex. IL-2(E52K) and wildtype IL-2 bound to hCD25 with similar affinity and induced STAT5 phosphorylation with similar potency; however, IL-2(E52K) did not trigger MACS. Thus, IL-2(E52K) serves as a molecular probe that can decouple canonical and MACS IL-2 signaling mediated *via* certain chemokine receptors. We find that in addition to CCR7, CXCR4 and CCR5 can also associate with hCD25 in the presence of 7G7B6 and IL-2, and all three chemokine receptor–CD25 complexes can trigger anti-CD25– and HS-dependent integrin activation. Furthermore, we report that 7G7B6 did not impair IL-2-induced STAT5 phosphorylation, suggesting that MACS complements canonical IL-2 signaling. Indeed, CD122 and CCR7 are present in the same anti-CD25–induced multiprotein complex, where they can initiate two coincident IL-2-initiated signals.

The results of this work indicate that IL-2 MACS is initiated by the interaction of IL-2-bound CD25 with a chemokine receptor. Previous work showed that the antimouse CD25 antibody PC61 triggers CD25–CCR7 association and integrin activation but did not establish that this association caused the signaling event. Here, we report that an IL-2 mutant that prevents CD25–CCR7 complex formation blocks integrin activation, thereby showing that MACS requires the complex. CD25–CCR7 complex formation requires IL-2–CD25 interaction because a CD25 mutant that disrupts the IL-2 binding site prevents assembly of the anti-CD25–induced CD25–IL-2–CCR7 complex (8). Accordingly, both complex formation and integrin activation are inhibited by an anti-IL-2 antibody that blocks IL-2 binding to CD25 (8). IL-2(E52K) was designed to spare the interaction sites for the αβγ IL-2 receptor (Fig. 1). We confirm that it maintains IL-2 binding to CD25 and JAK/STAT5-mediated canonical signaling (Fig. 2, *C* and *D*). In Jurkat cells, integrin activation is inhibited by silencing CCR7 and blocked by pertussis toxin (8), indicating that CCR7 generates the activation signal. IL-2 Glu^52^ is on the opposite face of IL-2 from the αβγ receptor interaction sites (Fig. 1), and IL-2(E52K) disrupts CD25–IL-2–CCR7 complex formation. Therefore, the interaction of this face of IL-2 with the chemokine receptor may contribute to complex formation and resulting integrin-activating signals. Thus, IL-2 may serve as part of a bridge between CD25 and a chemokine receptor, possibly by directly interacting with both receptors in a ternary complex. Future structural studies will elucidate the molecular details of the formation of this new IL-2-dependent signaling complex.

Multiple chemokine receptors that recognize distinct ligands can participate in MACS. Previous studies suggested that both CCR7 and CXCR4 could contribute to integrin activation induced by IL-2 in the presence of PC61 (8). We now find that in addition to CCR7, CXCR4 and CCR5 can enter IL-2-dependent complexes in the presence of an anti-CD25 antibody (Fig. 3*D*). Importantly, chemokine receptors can form oligomers in which negative binding cooperativity takes place between the binding pockets of these receptors, causing receptor-specific antagonists to cross-inhibit signaling in these oligomers (23). Thus, it is difficult to ascertain which specific chemokine receptors might directly bind to IL-2-occupied CD25. CCR7 and CXCR4 can associate with IL-2-bound CD25 in HEK293T cells that express little CCR5. Furthermore, CCR5, in contrast to CXCR4 and CCR7, will associate with CD25 in the presence of IL-2(E52K), indicating that this association does not depend on the presence of CCR7 or CXCR4 in the complex. Maraviroc, an antagonist of CCR5 (21), blocks the IL-2(E52K)-dependent CD25–CCR5 complex and inhibits the capacity of IL-2(E52K) to support 7G7B6-induced integrin activation, indicating that CCR5 can generate the activation signal. Thus, it seems likely that multiple chemokine receptors can interact with IL-2-bound CD25; these receptors have differing ligand recognition specificities (24, 25), suggesting a distinct mode of interaction for each chemokine receptor. In sum, both CCR7 and CCR5 chemokine receptors can form 7G7B6-induced complexes with IL-2-bound CD25 to generate integrin activation signals. MACS can stimulate the suppressive function of Tregs that express CD25 and chemokine receptors (8). In addition to Tregs, many different cell lineages can express CD25 (26, 27, 28) and possess distinct and varying chemokine receptor repertoires (24). Thus, these data suggest that MACS may affect the function of diverse cell types.

HS is a physiological ligand for IL-2 and can enhance IL-2-induced integrin activation (8). We report that HS-induced integrin activation is associated with the formation of an hCD25–CCR7 complex, and disruption of this complex by the IL-2(E52K) mutation blocks integrin activation. Thus, HS-triggered MACS can be ascribed to HS, enabling IL-2 to mediate assembly between CD25 and CCR7. These results indicate that HS and certain anti-CD25 antibodies, two chemically distinct moieties, depend on a common feature of IL-2 to trigger MACS. Furthermore, a detailed understanding of the structural requirements for HS-induced MACS could have important implications for variations in HS structure in different tissues and in different physiological and pathological settings.

Our findings demonstrate that canonical and MACS IL-2 signaling can operate in concert. Consistent with the lack of effect of heparin on IL-2-dependent cell proliferation, we observed that HS (*M*_r_ ∼30,000) at ∼100-fold greater molar concentration than IL-2 did not impair IL-2-induced STAT5 phosphorylation in IL-2Rα^+^ YT-1 cells (Fig. 5*D*). Furthermore, the failure of soluble HS to reduce IL-2-induced STAT5 phosphorylation may suggest that IL-2 binding to cell surface HS does not contribute to canonical IL-2 signaling. Furthermore, we quantified 7G7B6 binding to CD25 in IL-2Rα^+^ YT-1 cells and found that saturating concentrations did not impair IL-2-induced STAT5 phosphorylation, consistent with its known lack of effect on IL-2 binding to the receptor (17). The fact that both HS and 7G7B6 induce MACS without impairing canonical IL-2 signaling shows that both pathways can operate in coincidence and suggests the possibility that a single protein complex contains receptors that initiate both pathways. When 7G7B6 was saturating, that is, when all CD25 was occupied by 7G7B6, we found that CD122 and CCR7 were physically associated, that is, in the same 7G7B6-induced IL-2-dependent protein complex. Furthermore, this association is lost in the presence of IL-2(E52K), which dissociates CCR7 from the CD25–CCR7 complex. Thus, although we had depicted MACS as an alternative to canonical IL-2 signaling (8), these data suggest the formation of a protein assembly that can initiate two distinct IL-2 signaling pathways, both of which can contribute to Treg function.

## Experimental procedures

### Reagents and antibodies

Anti-CCR7 polyclonal antibody (Novus Biologicals; catalog no.: NBP2-67324), anti-HA mouse monoclonal antibody (12CA5), anti-HA mouse monoclonal antibody (BioLegend; catalog no.: 900801), anti-HA rabbit polyclonal antibody (BioLegend; catalog no.: 923501), biotin–antihuman CCR7 mouse monoclonal antibody (BioLegend; catalog no.: 353240), biotin–anti-hCD25 goat polyclonal antibody (R&D Systems; catalog no.: BAF223), biotin–antimouse CD25 goat polyclonal antibody (R&D Systems; catalog no.: BAF2438), antimouse CD25 monoclonal antibody (clone PC-61.5.3, BioXcell; catalog no.: BE0012), anti–hCD25 monoclonal antibody (7G7B6) (17), anti–hCD25 monoclonal antibody (BC96, BioLegend; catalog no.: 302609), streptavidin–horseradish peroxidase (Invitrogen; catalog no.: S911), IRDye 800CW Goat Antimouse immunoglobulin G (IgG) Secondary Antibody (LI-COR; catalog no.: 926-32210), IRDye 800CW Goat Anti-Rabbit IgG Secondary Antibody (LI-COR; catalog no.: 926-32211), IRDye 680RD Goat Anti-Rabbit IgG Secondary Antibody (LI-COR; catalog no.: 926-68071), IRDye 680RD Goat Antimouse IgG Secondary Antibody (LI-COR; catalog no.: 926-68070), and Protein G Agarose (GenScript; catalog no.: L00209). HS sodium salt from bovine kidney was from Sigma–Aldrich (catalog no.: H7640).

### Cell culture

HEK293T cell lines expressing HA-tagged mouse CD25 or hCD25 were constructed and cultured as described (29). IL-2 Rα^+^ YT-1 cells were derived from YT-1 cells (20), induced to express endogenous CD25, as described previously (19).

### ELISA

The formation of the CD25–CCR7 complex was assayed by ELISA as previously described (8). Briefly, microplate wells were coated with anti-HA antibody, and HEK293T cells expressing HA-CD25 were added to the plate and stimulated with 5 μg/ml PC61 and 1 μg/ml IL-2 for 1 h at 37 °C. Cells were lysed, and the plate was then washed, and bound CCR7 or CD25 was labeled with biotinylated primary antibodies, and after washing, bound antibodies were complexed with streptavidin–horseradish peroxidase followed by chemiluminescence substrate (Thermo Scientific; catalog no.: 32106). Chemiluminescence intensity was assessed by a microplate scanner (Spark; Tecan Life Sciences) to measure bound CCR7. In subsequent experiments, bound anti-CD25 or CCR7 were assessed by SDS-PAGE followed by immunoblotting.

### Measurement of IL-2 binding to CD25

The 6x-His-tagged extracellular domain of hCD25 (amino acid residues: 1-217), human IL-2, and IL-2(E52K) were expressed in Expi293 cells (Thermo Fisher) and purified using His•Bind Resin (EMD Millipore). Microplates (Greiner Bio-One) were coated with hCD25 (2 μg/ml) in carbonate/bicarbonate coating buffer (pH 9.2) and blocked with 2% bovine serum albumin. Increasing concentrations of IL2 or IL-2(E52K) were added, incubated for 2 h at room temperature, and then washed. Subsequently, the bound IL-2 was quantified using the ELISA MAX Deluxe Set Human IL-2 (BioLegend).

### Subcellular fractionation, coimmunoprecipitation, and immunoblot analysis

Membrane fractions from HA-CD25–expressing HEK293T cells were prepared by differential centrifugation as described (30). Cell membranes were washed and then suspended in fractionation buffer containing 1% Nonidet P-40 on ice for 1 h. The solubilized membrane preparations were incubated with 5 μg/ml HS at 37 °C for 30 min and incubated with anti-HA antibody immobilized on protein G beads in the lysis buffer at 4 °C for 2 h to overnight to capture HA-CD25. The isolated protein complex was washed with the lysis buffer and subjected to SDS-PAGE. Bound proteins were detected by immunoblotting with designated antibodies.

### Measurement of G protein–coupled receptor association with CD25–CCR7 complex

Constructs encoding designated EGFP-3xFLAG-tagged mouse G protein–coupled receptors (GPCRs) were generated by cloning 3xFLAG-tagged GPCR sequences into the pEGFP-C1 vector (Takara Bio). HEK293T cells stably expressing HA-tagged mouse CD25 were transfected with these EGFP-3xFLAG-GPCR constructs using polyethyleneimine. After 24 h of incubation, cells were stimulated for 30 min at 37 °C with PC61 (5 μg/ml) and mouse IL-2 (1 μg/ml). Cells were then lysed on ice for 15 min using lysis buffer, and the soluble fraction was obtained by centrifugation at 12,000 rpm for 15 min. The resulting lysates were incubated overnight at 4 °C with anti-FLAG antibody resin (L00432; GenScript). Bound proteins were washed three times with lysis buffer and resolved on SDS-PAGE. Coprecipitated CD25 and GPCRs were detected by immunoblotting using an anti-HA polyclonal antibody (BioLegend; catalog no.: 902301) and a biotinylated anti-FLAG antibody (GenScript; catalog no.: A01429). Band intensities were quantified using an LI-COR Odyssey CLx infrared imaging system (LICORbio).

### Isolation and activation of human primary CD4^+^ T lymphocytes

Human blood was obtained from normal adult human volunteers. Red blood cells from the acquired whole blood were lysed with ACK lysis buffer (Gibco; catalog no.: A1049201), followed by magnet separation using the human whole blood CD4+ T-cell negative isolation kit (BioLegend; catalog no.: 480162). For soluble ligand-binding assays with freshly isolated CD4+ T lymphocytes, cells were labeled with PerCP-hCD4 (BioLegend; catalog no.: 357414), FITC-hCD127 (BioLegend; catalog no.: 351312), and PE-hCD25 (BioLegend; catalog no.: 302606) to distinguish CD25^hi^CD127^lo^ Tregs and Tconv.

### Soluble ligand binding assay

Cells were plated in a 96-well plate and incubated with the following ligands for 30 min at 37 °C in Hanks' balanced salt solution (HBSS) with Ca/Mg (Gibco; catalog no.: 14-025-092): 5 μg/ml human MAdCAM-1 Fc (R&D Systems; catalog no.: 6056-MC) for YT-1 hCD25 cells or human VCAM-1 Fc (R&D Systems; catalog no.: 862-VC) with human primary CD4^+^ T cells, 0.5 μg/ml IL-2, 10 μg/ml IgG control or 7G7B6, 5 μg/ml HS, 200 ng/ml pertussis toxin, 100 nM phorbol 12-myristate 13-acetate (Sigma; catalog no.: P8139), and 10 mM EDTA (Invitrogen; catalog no.: AM9260G). Cells were then immediately washed with 1X HBSS with Ca/Mg, followed by incubation with APC-antihuman IgG Fc (1:25, BioLegend; catalog no.: 410714) for 1 h on ice. Cells were washed in 1X HBSS with Ca/Mg, and geometric mean fluorescence intensity (MFI) was determined on an Accuri C6+ flow cytometer (BD Biosciences). Geometric MFI of EDTA groups was subtracted from MFI without EDTA groups to report ligand binding (MFI ΔEDTA). Triplicate measurements were made in each experiment, and each experiment was independently replicated at least three times with similar results.

### STAT5 phosphorylation

Cells were plated in each well of a 96-well plate and incubated with indicated concentrations of IL-2, 5 μg/ml isotype or 7G7B6, or 5 μg/ml HS in 1X PBS. Cells were stimulated for 30 min at 37 °C and immediately fixed and permeabilized using the Transcription Factor Phospho Buffer Set (BD Biosciences; catalog no.: 563239). Cells were then washed and incubated with APC-anti STAT5 (1:50 dilution, BD Biosciences; catalog no.: 612599) for 1 h on ice in the wash buffer. Cells were then washed twice, once with the wash buffer and next with 1X PBS, and MFI was determined on an Accuri C6+ flow cytometer. Triplicate measurements were made in each experiment, and each experiment was independently replicated at least three times with similar results.

### Quantification of CD122 association with CD25–CCR7 complex

FLAG-tagged human CCR7 was PCR amplified and cloned into the BamHI and EcoRI restriction sites of the lentiviral pLVX-Het1 vector (Takara Bio; catalog no.: 635075). The resulting plasmid was transfected into HEK293T cells using polyethyleneimine (Polysciences; catalog no.: 24765), followed by a 48-h incubation. Viral supernatant was collected and concentrated by Lenti-X concentrator (Takara Bio; catalog no.: 631231) overnight at 4 °C. The pelleted virus, resuspended in PBS, was used to transduce IL-2Rα^+^ YT-1 cells in the presence of 8 μg/ml polybrene and centrifuged at 2500 rpm for 30 min to enhance transduction efficiency. Transduced cells were subsequently sorted by flow cytometry to isolate those expressing FLAG-tagged CCR7. Sorted cells were stimulated for 30 min at 37 °C with 7G7B6 (5 μg/ml) and IL-2 (1 μg/ml). Poststimulation, cells were lysed on ice for 15 min in a lysis buffer containing 50 mM Tris–Cl (pH 7.4), 150 mM NaCl, 0.5% NP-40, 0.5 mM CaCl_2_, 0.5 mM MgCl_2_, and an EDTA-free protease inhibitor cocktail (MedChemExpress; catalog no.: HY-K0011). Lysates were then centrifuged at 12,000 rpm for 15 min to remove insoluble debris. The resulting lysates were incubated in a 96-well microplate (Greiner Bio-One; catalog no.: 675074) coated with rabbit anti-FLAG antibody (MilliporeSigma; catalog no.: F7425) for 2 h at room temperature. After incubation, unbound components were removed by washing three times with buffer (0.05% Tween-20 in PBS), and associated CD122 or captured CCR7 was reacted with biotinylated anti-CD122 (G-Biosciences; catalog no.: ITA0042-B) or anti-CCR7 (BioLegend; catalog no.: 353240) antibodies, respectively. The biotinylated antibodies were detected by streptavidin–horseradish peroxidase followed by 3,3′,5,5′-tetramethylbenzidine chromogenic substrate (Thermo Scientific; catalog no.: 34021). The reaction was terminated using 2 M H_3_PO_4_, and the absorbance of the reaction product was measured at 450 nm using a Spark microplate scanner (Tecan Life Sciences).

### Statistical analysis

Statistical analysis was performed using PRISM software (version 10; GraphPad Software, Inc), and all datasets were checked for Gaussian normality distribution. Data analysis was performed using a two-tailed *t* test, one-way ANOVA, or two-way ANOVA followed by a Bonferroni post-test as indicated in the legends to the figures. The resulting *p* values are indicated as follows: NS, not significant, ∗*p* < 0.05; ∗∗*p* < 0.01; ∗∗∗*p* < 0.001; and ∗∗∗∗*p* < 0.0001. Plotted data are the mean ± SEM of at least three independent experiments.

## Data availability

Data are available from the corresponding author upon request.

## Supporting information

This article contains supporting information.

## Conflict of interest

The authors declare that they have no conflicts of interest with the contents of this article.
