# Supplementary material for: A CD25–chemokine receptor complex initiates noncanonical IL-2 signaling
Source: J Biol Chem. 2025 Nov 25;302(1):110981. doi: 10.1016/j.jbc.2025.110981 (PMC12810549; doi:10.1016/j.jbc.2025.110981)
Supplement: Figure S1 [file mmc1.pdf]

Figure S1

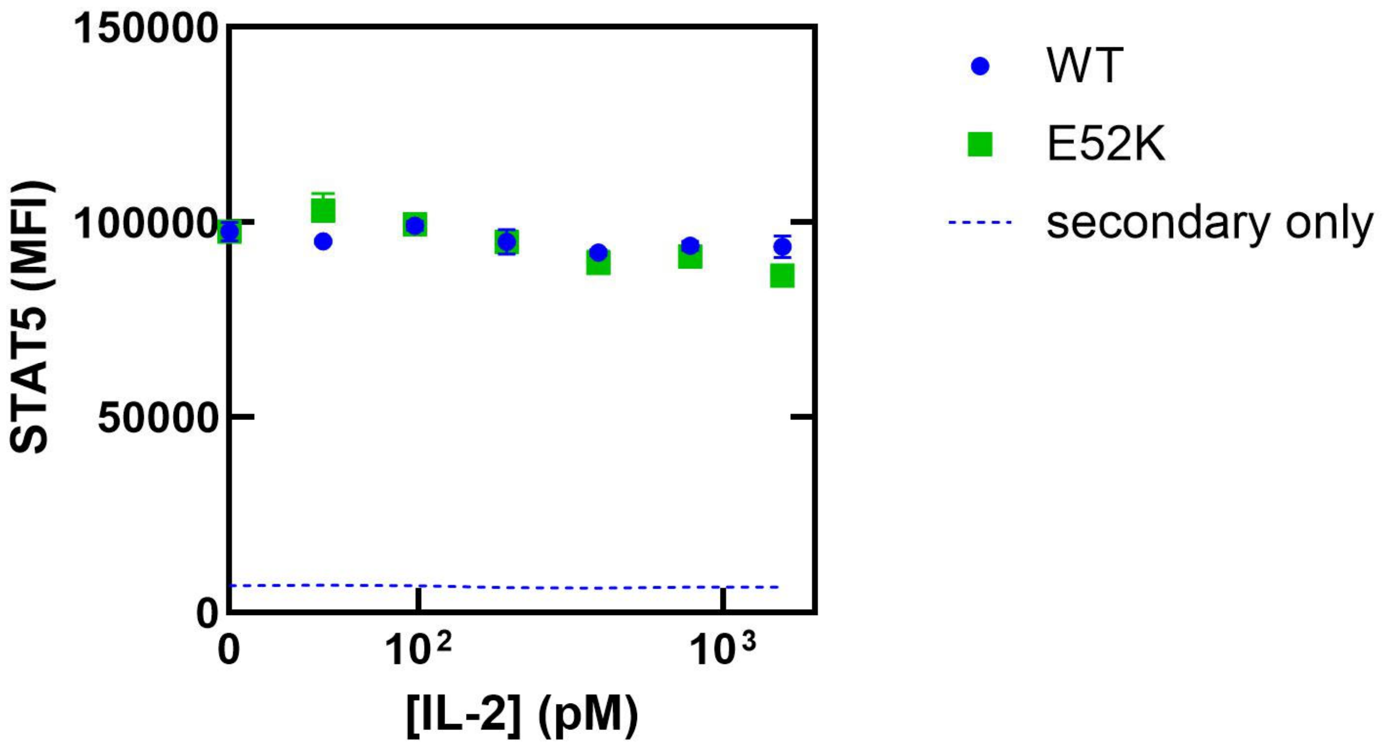

Figure S1 IL-2 does not change the total STAT5 expression. Total STAT5a/b (STAT5) was quantified in parallel with pSTAT5 responses of IL2Rα YT-1 cells (Fig. 2D). IL2Rα YT-1 cells were stimulated with varying concentrations of IL-2 WT (WT, blue) or IL-2(E52K) (E52K, green) for 30 min at 37 C, followed by staining with anti-total STAT5 and FITC-anti-rabbit IgG, and analysis by FACS.
